# Rapid Thermal Processing to Enhance Steel Toughness

**DOI:** 10.1038/s41598-017-18917-3

**Published:** 2018-01-11

**Authors:** V. K. Judge, J. G. Speer, K. D. Clarke, K. O. Findley, A. J. Clarke

**Affiliations:** Colorado School of Mines, George S. Ansell Department of Metallurgical and Materials Engineering, Advanced Steel Processing and Products Research Center, 1500 Illinois St., Golden, CO 80401 USA

## Abstract

Quenching and Tempering (Q&T) has been utilized for decades to alter steel mechanical properties, particularly strength and toughness. While tempering typically increases toughness, a well-established phenomenon called tempered martensite embrittlement (TME) is known to occur during conventional Q&T. Here we show that short-time, rapid tempering can overcome TME to produce unprecedented property combinations that cannot be attained by conventional Q&T. Toughness is enhanced over 43% at a strength level of 1.7 GPa and strength is improved over 0.5 GPa at an impact toughness of 30 J. We also show that hardness and the tempering parameter (TP), developed by Holloman and Jaffe in 1945 and ubiquitous within the field, is insufficient for characterizing measured strengths, toughnesses, and microstructural conditions after rapid processing. Rapid tempering by energy-saving manufacturing processes like induction heating creates the opportunity for new Q&T steels for energy, defense, and transportation applications.

## Introduction

As-quenched, martensitic steel is strong, but often notably brittle. In order to increase ductility and toughness, martensite is heat-treated by a process called tempering^1–3^. Strength typically decreases with increasing tempering temperature and time, and a corresponding increase in toughness is expected. However, there is an established decrease in toughness in medium carbon, low-alloyed steels for tempering times of 1 h with increasing temperature between 200 and 400 °C^2,4–13^. This phenomenon, known as tempered martensite embrittlement (TME), has been attributed to a multitude of mechanisms, including: the thermal and mechanical decomposition of retained austenite^6,12^, interlath brittle cementite formation^6^, and cementite particle growth^7,13^. The severity of TME is often characterized by the extent of the observed loss of toughness. Tempered martensite embrittlement leads steel manufacturers and end-users to avoid tempering in the affected time-temperature regime, thereby eliminating certain strength-toughness combinations that would be desirable if suitable heat treatments could be designed.

Short-time tempering at high temperatures (500 to 700 °C) has recently been suggested to improve impact toughness via carbide (cementite) refinement^14–17^. The time-scale of these rapid tempering heat treatments is said to limit dislocation recovery, thereby providing increased nucleation sites for the formation of fine, dispersed cementite particles. Here, we investigate the effects of short-time tempering within a lower tempering temperature range. Not only is TME active within the explored tempering regime, but different mechanisms are expected to dominate the microstructural evolution, and hence toughness, relative to high temperature tempering^18–20^. Older work^21–23^ began to explore rapid tempering within the TME regime; however, the current study reveals the novel discovery of improved toughness for a given strength level compared to conventional tempering treatments.

**Figure 1:**
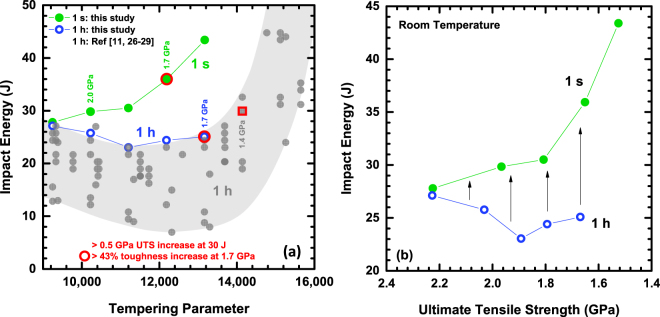
Comparison of room temperature impact energy (J) for conventional (1 h) and short-time (1 s) tempering conditions as a function of (**a**) tempering parameter (TP) and (**b**) ultimate tensile strength, or UTS (GPa). All (**a**) UTS values are rounded to the nearest 0.1 GPa. Referenced data corresponds to quenched and tempered 4340 steel.

Figure 1 illustrates the effects of short-time tempering on impact toughness with respect to tempering parameter (Fig. 1a) and ultimate tensile strength, or UTS (Fig. 1b). Tempering parameter (TP) is a metric that is often used in industry to equate tempering treatments that utilize different operating times and temperatures, where tempering treatments are considered to be equivalent if they possess the same tempering parameter value. Figure 1a highlights the marked improvement in strength-toughness performance achieved via rapid tempering in the context of a wide range of conventionally tempered 4340 steels^11,24–27^. The utilization of short-time tempering is shown to improve impact toughness by over 43% at a strength level of 1.7 GPa and enhances strength by more than 0.5 GPa at a constant toughness of 30 J. Additionally, the conventional tempering condition of 1 h exhibits classic TME through a significant decrease in toughness within the TP range of 9,000 to 11,000, while the TME “trough” is shown to diminish with short-time tempering. Figure 1b further illustrates the relationship between strength and impact toughness for short-time and conventionally tempered conditions. Overall, a consistent improvement in toughness and a reduction in TME with short-time tempering is observed within the entirety of the TME regime.

**Figure 2:**
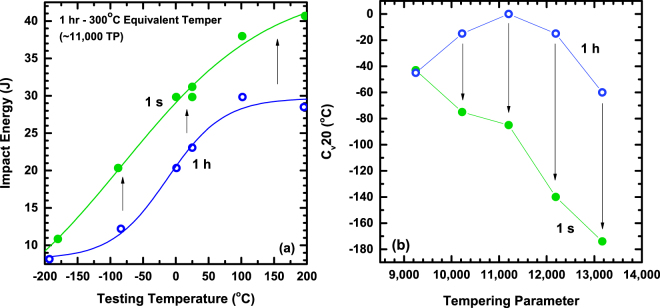
Toughness behavior of 1 s and 1 h tempering conditions for testing temperatures ranging from −200 to 200 °C. (**a**) Ductile-to-brittle-transition curves for the 11,000 TP condition, comparing 1 s and 1 h tempering treatments for various testing temperatures. (**b**) Index temperature, C_V_ 20, for conventional and short-time tempering conditions over a range of tempering parameters. Lower values of C_V_ 20 represent superior toughness.

To gain further insight, the Charpy impact toughness behavior at various temperatures was measured. Figure 2a and b show the behavior of the 1 h and 1 s tempering treatments for various testing temperatures. Figure 2a displays the impact toughness for two time conditions at a TP of 11,000, corresponding to a 1 h at 300 °C conventional tempering treatment. Toughness not only improves under the short-time conditions at room temperature, but also consistently improves across the full range of testing temperatures. The temperature correlating to a fracture energy of 20 Joules (C_v_ 20) was used as the criterion to compare the ductile-to-brittle-transition-temperature (DBTT) of the Charpy V-notch (C_v_) impact toughness results for the TPs studied, where a lower C_v_ 20 temperature indicates superior toughness. Figure 2b shows the results comparing the short-time and conventional C_v_ 20 temperatures. The manifestation of TME is clearly observed for the 1 h tempering condition via an increase in C_v_ 20 temperature from 9,000 to 11,000 TP, consistent with the room temperature toughness data (Fig. 1a). Conversely, short-time tempering leads to overall lower C_v_ 20 temperatures and a more linear decrease in C_v_ 20 temperature with increasing TP, signifying significant improvements in toughness. A slight plateau is observed from 10,000 to 11,000 TP, indicating the diminished TME trough.

**Figure 3:**
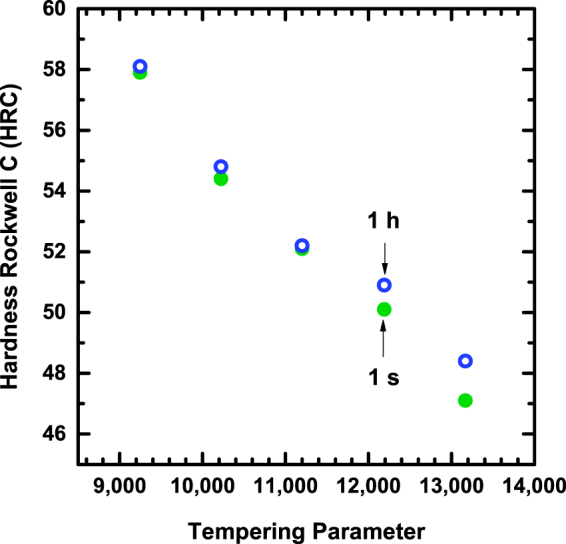
Hardness (Rockwell C scale) of 1 h and 1 s tempering treatments for equivalent tempering parameters (TPs).

Many of the present results have been presented in terms of tempering parameter. As mentioned, two tempering treatments with varying times and temperatures are considered to be equivalent if the same tempering parameter (and hardness) value is produced. This concept was first proposed by Hollomon and Jaffe, with the tempering parameter represented by^28^:

1 $${\rm{TP}}={\rm{T}}[\mathrm{log}({\rm{t}})+{\rm{c}}]$$

where T is absolute temperature, t is tempering time, and c is a constant that varies for the steel being treated. The tempering parameter has become ubiquitous in the field of steel metallurgy and it is now widely accepted that equivalent tempered hardness values for a given steel imply an equivalent degree of tempering and similar mechanical behavior. The tempering conditions evaluated in this study were designed to yield the same hardness value for a given degree of tempering in a medium-carbon, low-alloyed steel, as displayed in Fig. 3. Based on the Hollomon-Jaffe tempering parameter, the 1 s and 1 h tempering treatments are considered to impart the same degree of tempering for a given tempering parameter, and would therefore be expected to demonstrate comparable mechanical behavior. However, as demonstrated in Figs 1 and 2, the two time conditions exhibit markedly different toughness behavior for a given tempering parameter. While the tempering parameter is an acceptable method for producing tempered steels with equivalent hardness values, it is clear that it does not encompass and accurately equate the microstructural processes and resultant toughness and strength properties that occur during tempering.

**Figure 4:**
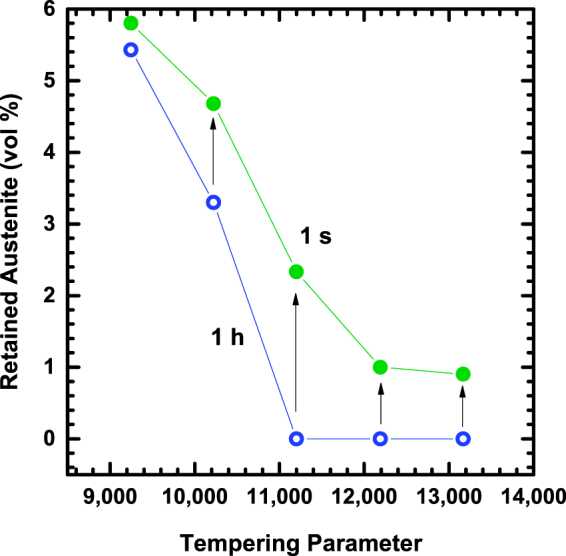
Comparison of retained austenite contents for 1 s and 1 h TPs measured by x-ray diffraction.

The mechanical properties presented here demonstrate the significant advantages associated with short-time thermal processing. Additionally, it is suggested that the long-accepted Hollomon-Jaffe tempering parameter, based on equivalent hardness, is not a comprehensive method to equate tempering processes, particularly when short-time tempering is considered. More work is needed to clarify the microstructural mechanisms responsible for the effect of rapid tempering on TME and toughness, which will need to be incorporated into a new model for predicting the degree of tempering. The early stage of this effort is shown in Fig. 4, where differences in retained austenite content between short-time and conventional tempering conditions highlight assorted microstructures at equivalent TPs and hardness levels. The decreased susceptibility of the short-time tempering conditions to TME and the higher observed amount of retained austenite are consistent with the hypothesis that TME is caused by austenite decomposition into ferrite and interlath cementite during tempering. The mechanisms contributing to the overall improvement in toughness with short-time tempering may also be related to the details associated with austenite decomposition, but further evaluation is required to develop a complete understanding.

The observations of hardness and microstructure (retained austenite) in the present work demonstrate that different tempering mechanisms influence hardness and toughness, and these mechanisms do not obey the same relationship for time-temperature equivalence. Such mechanisms include carbon diffusion in (body-centered cubic or tetragonal) martensite, carbon diffusion in (face-centered cubic) austenite, and iron self-diffusion. It has been recently pointed out^29^ that these fundamental mechanisms are not incorporated in the commonly used Hollomon-Jaffe tempering parameter. The present results confirm the critical relevance of this concern, as well as the opportunity to develop new fundamental kinetic models for retained austenite decomposition and martensite tempering.

While tempering temperatures between 200 and 400 °C have historically been avoided in steel processing due to the production of inferior toughness properties, short-time tempering provides a pathway to temper within this strength regime to achieve desirable mechanical property combinations. These findings provide a processing breakthrough to not only diminish TME, but also substantially improve toughness in steels tempered within the previously avoided TME regime. Furthermore, rapid tempering can be achieved by already established processing methods; for example, induction heating has the potential to save significant time and energy^30^, while producing steels with superior properties compared to conventionally tempered steels. The application of short-time tempering at relatively low tempering temperatures to achieve improved mechanical behavior through enhanced microstructural design presents an opportunity to significantly impact steel manufacturing and user communities, benefiting mankind as a whole by producing superior steel products for everyday and high-performance applications at a lower cost to the environment.

## Methods

The steel used in this study was from an industrial heat produced by ArcelorMittal, received in the form of 12.7 mm (0.5 in) plate, courtesy of Los Alamos National Laboratory (Los Alamos, NM, USA). The chemical composition is provided in Table 1. The plate was sectioned into 11 × 11 × 90 mm rectangular blanks and subjected to a series of heat treatments. The blanks were first austenitized in a vacuum furnace at 845 °C for 1 h, then oil quenched to produce a martensitic microstructure.

**Table 1:** Chemical Composition of AISI 4340 Research Material (wt pct.).

C	Mn	Si	Ni	Cr	Mo	Nb	V	Al	S	P	Cu
0.41	0.71	0.25	1.76	0.75	0.26	0.005	0.047	0.008	0.001	0.009	0.14

Base temperatures of 200, 250, 300, 350, and 400 °C were chosen for a tempering time of 1 h, due to the known appearance of TME within this temperature regime. Tempering parameters were then calculated with a c-value of 16 for the 1 h treatments and corresponding tempering temperatures were determined for the “equivalent” 1 s treatments. The blanks were tempered at temperatures ranging from 200 to 550 °C at tempering times of 1 s or 1 h. The specific tempering temperatures are provided in Table 2. The short-time tempers at 1 s were conducted with a Gleeble^**®**^ 3500 thermo-mechanical simulator with resistive heating and helium quenching, while the 1 h tempering treatments were performed using liquid salt baths and water quenching. The temperature of the bath was monitored for all 3600 s tempers, while the temperature was monitored via a thermocouple spot-welded to the surface of each sample for the Gleeble^**®**^ 3500 tempering.

**Table 2:** Time-Temperature Tempering Matrix.

Time	Temperature (°C)				
1 h	200	250	300	350	400
1 s	305	366	427	489	550

Thermal profiles of the short-time tempering operations were recorded and used to calculate an equivalent isothermal tempering time for the target tempering temperatures outlined in Table 2. This was achieved by converting the actual time-temperature profiles of the Gleeble^**®**^ 3500 tempering processes (heating and cooling included) to equivalent tempering parameters utilizing a modified tempering parameter model^31^. In this model, the tempering behavior is considered “additive” and thus the tempering parameter is numerically integrated over short “isothermal” time steps. This is accomplished by equating the tempering parameters of each process:

2 $$T(c+\,{log}\,{\rm{\Delta }}{t})={T}^{\ast }(c+\,{log}\,{\rm{\Delta }}{t}^{\ast })$$

where c is the c-value and T* and t* are the temperature and time, respectively, of the equivalent isothermal tempering cycle. T and t are the tempering and time, respectively, of the non-isothermal, actual tempering cycle. T* was set equal to the peak (target) temperature of the non-isothermal cycle. The time of the equivalent isothermal cycle, t*, was determined by solving for Δt* at each increment of the cycle and then summing all Δt* values. The largest recorded tempering time deviation associated with the 1 s tempering treatments was 0.2 s. This corresponds to a calculated hardness deviation of 0.2 HRC and tempering parameter deviation of 60, and was considered acceptable.

Vickers microhardness measurements were performed on polished sections of the tempered specimens using a 500 gmf load and a 10 s dwell time. The Rockwell C (HRC) hardness values reported here were obtained with the conversion capabilities of a LECO MHT200 microhardness indenter. The remainder of the rectangular blanks were machined into standard^32^ Charpy V-notch specimens to measure impact toughness. Charpy testing was conducted in accordance with the standard ASTM E23, with the exception of samples tested at a temperature of 200 °C^32^. Samples tested at 200 °C were heated via a solid-state thermal mass within an at-temperature furnace, as opposed to the standard gas and liquid media, and were determined to reach thermal equilibrium after 5 minutes.

Uniaxial tensile testing was carried out on an MTS 22-kip hydraulic frame equipped with V-wedge grips. Displacement was measured using a 10 mm extensometer with a strain rate of 0.015 mm/mm/min. If the extensometer reached its limit of 15%, then the program was stopped and the extensometer reset. Prior to testing, five diameter measurements were taken along the reduced cross-section within the specified extensometer range. The five measurements were averaged for engineering stress calculations, while the change in gage length given by the extensometer was used for engineering strain calculations. Ultimate tensile strength (UTS) was taken to be the highest stress value.

X-ray diffraction samples were prepared by sectioning specimens and lightly grinding the appropriate surface with a progression of 250, 320, 400, and 600 grit metallographic paper. Samples were subsequently thinned in a solution of 10 parts deionized water, 10 parts hydrogen peroxide, and 1 part hydrofluoric acid for 15 to 30 minutes to achieve a thickness reduction of at least 0.005 in., per ASTM standard E975^33^. It is important to recognize the limitations associated with measuring low percentages of retained austenite using XRD. There have been varying reports on the minimum amount of retained austenite reliably detected by XRD, ranging from ~1.5 to 2%^33,34^. Additionally, the calculated amount of retained austenite at small percentages can be significantly affected by peak fitting operations. Four measurements were taken per condition to assess the variability in the measurements. The standard deviation of the measurements went from approximately 0.1 to 1.8 vol. % retained austenite depending on the condition. The short-time conditions tended to exhibit more variability in measurements. Therefore, it is of interest to explore more accurate techniques for measuring small amounts of retained austenite in the future, which will include Mossbauer effect spectroscopy. For the purposes of this study, the retained austenite results are utilized to relate microstructural trends with properties, as these data are the primary source of information related to microstructure.

Retained austenite volume fractions were determined using nickel-filtered copper radiation and a Phillips X-pert diffractometer with operating conditions of 45 kV and 40 mA. The diffractometer was instrumented with an X’celerator detector and 1° slit. Four ferrite/martensite peaks ({110}, {200}, {211}, {220}) and four austenite peaks ({111}, {200}, {220}, {311}) were compared to determine the amount of retained austenite. Samples were scanned at a 2θ range of 40 to 105°, with a step size of 0.05° and 200 s dwell time. Retained austenite values were calculated in accordance with the SAE method^35^.
