# Publisher Correction: Rapid Thermal Processing to Enhance Steel Toughness

**DOI:** 10.1038/s41598-020-73765-y

**Published:** 2020-10-15

**Authors:** V. K. Judge, J. G. Speer, K. D. Clarke, K. O. Findley, A. J. Clarke

**Affiliations:** Colorado School of Mines, George S. Ansell Department of Metallurgical and Materials Engineering, Advanced Steel Processing and Products Research Center, 1500 Illinois St., Golden, CO 80401 USA

Correction to: *Scientific Reports* 10.1038/s41598-017-18917-3, published online 11 January 2018

This Article contains errors in Figure 1a.

The red square used to denote > 0.5 GPa UTS increase at 30 J and the grey circle used to denote referenced data points were omitted from the key. Furthermore, the 1h reference numbers were incorrectly given as “11, 26-29”.

The correct version of Figure 1a appears below.

**Figure 1:**
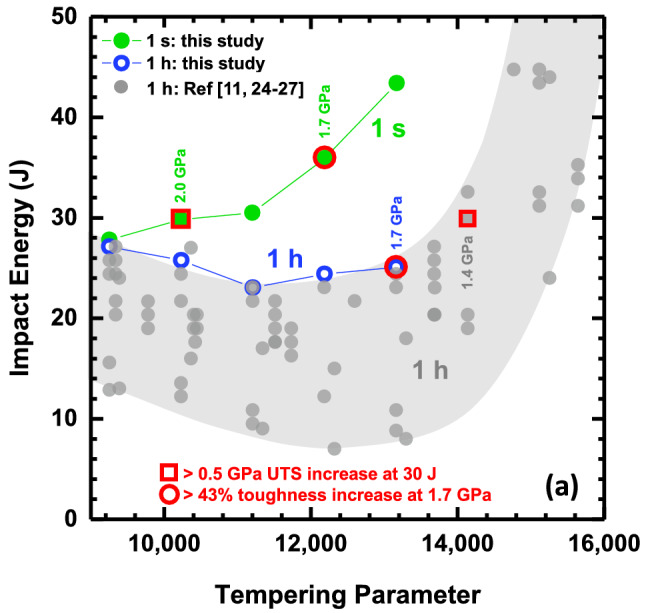
Comparison of room temperature impact energy (J) for conventional (1 h) and short-time (1 s) tempering conditions as a function of (**a**) tempering parameter (TP) and (**b**) ultimate tensile strength, or UTS (GPa). All (**a**) UTS values are rounded to the nearest 0.1 GPa. Referenced data corresponds to quenched and tempered 4340 steel.

